# Beyond the scope: endoscopic ultrasound—assisted appendicoscopy by use of a single-use cholangioscope for endoscopic appendicitis therapy: a case report

**DOI:** 10.1016/j.igie.2025.03.010

**Published:** 2025-04-02

**Authors:** Yu-Cong Chen, Yu-Chieh Weng, Yang-Bor Lu

**Affiliations:** 1 Department of Digestive Disease and, Hua Qiao University, Fujian, China; 2 Endoscopy Center, Xiamen Chang Gung Hospital, Hua Qiao University, Fujian, China

## Abstract

Endoscopic appendicitis therapy (EAT) is an emerging minimally invasive alternative to surgery. This case highlights using endoscopic ultrasound (EUS) to facilitate cholangioscope-guided appendicoscopy. A 41-year-old woman presented with acute right lower quadrant pain and leukocytosis. Computed tomography confirmed acute appendicitis. The patient opted for EAT. EUS identified appendiceal orientation, enabling smooth cholangioscope intubation via colonoscope. Irrigation cleared purulent discharge. Following rapid recovery, the patient remained asymptomatic at 3-month follow-up. EUS-assisted EAT improves access, precision, and efficiency in endoscopic management of appendicitis. This technique may broaden the role of minimally invasive, organ-preserving approaches as an alternative to surgery in selected patients.

Endoscopic appendicitis therapy (EAT) has emerged as a minimally invasive alternative to conventional surgery for acute appendicitis. This case report describes the novel use of endoscopic ultrasound (EUS) guidance to enhance the accuracy and efficiency of EAT. A 41-year-old woman presenting with acute appendicitis underwent EUS-assisted EAT, wherein a single-use cholangioscope was inserted through the therapeutic channel of a standard colonoscope. EUS imaging significantly facilitated precise identification of the appendiceal orientation, enabling smooth intubation and direct inspection of the inflamed mucosa. Repeated irrigation with saline solution and metronidazole successfully cleared pus and reduced inflammation. The patient experienced rapid pain relief, normalization of inflammatory markers, and an uneventful recovery. At 3 months, she remained asymptomatic without further intervention. This report highlights the potential of EUS-assisted EAT to overcome anatomic challenges and offer a minimally invasive alternative to surgery, especially for patients unsuitable for, or unwilling to undergo, traditional surgical approaches. The single-use video cholangioscope (VedVision, TY-ISS-L31; Vedkang, Changzhou, Jiangsu, China), which passes through the working channel of a duodenoscope, was originally designed for diagnosing and treating pancreaticobiliary diseases. Its utility has expanded to the inspection of intraluminal structures, such as the appendix, through standard therapeutic channel colonoscopes.^1^^,^^2^ A crucial aspect of therapeutic success in EAT is achieving effective intubation of the appendiceal orifice. In this report, we explore the use of EUS-assisted EAT to address this challenge.

As therapeutic endoscopy continues to evolve, the concurrent rise in the use of EUS has become a pivotal addition to clinical practice. EUS has significantly advanced the management of numerous conditions because of its versatile applications, including fine-needle aspiration and transmural drainage.^3^ Here, we describe a novel application of EUS-assisted EAT in the management of uncomplicated acute appendicitis, offering a minimally invasive alternative to traditional surgical approaches.

## Case presentation

**Figure 1:**
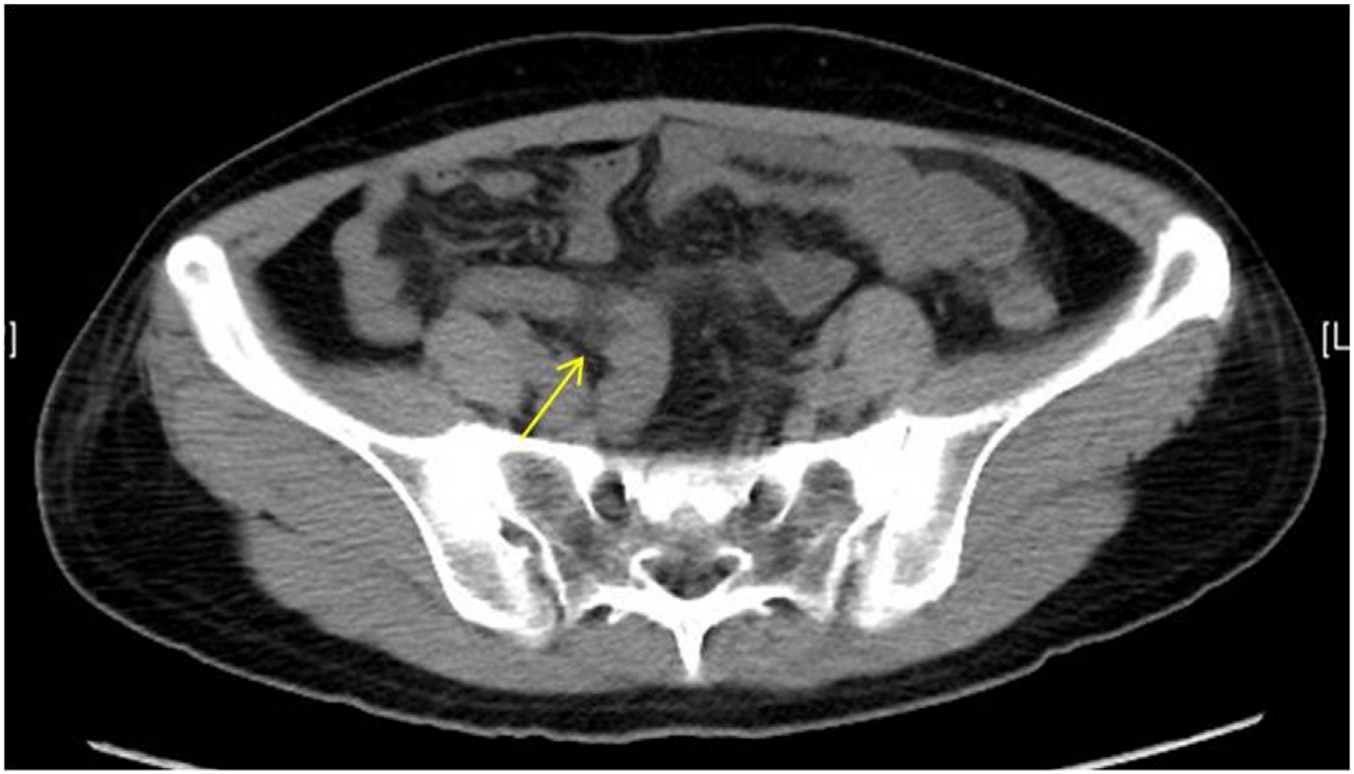
Noncontrast axial computed tomography of the abdomen showing increased appendiceal outer-to-outer diameter of 8.7 mm, wall thickening, and periappendiceal inflammation (*yellow arrow*). No evidence of free air or fluid to suggest perforation is observed.

A 41-year-old woman presented to the emergency clinic with acute periumbilical pain, accompanied by nausea and multiple episodes of vomiting. The colicky pain, exacerbated by movement, was her primary symptom. She had no significant medical, family, or social history. On physical examination, her abdomen was soft but tender in the right lower quadrant, with rebound pain. Laboratory tests revealed leukocytosis of 20.44 × 10^9^/L (reference range, 3.5-9.5 × 10^9^/L) and a neutrophil of 93% (reference range, 40%-75%). The high-sensitive C-reactive protein level was elevated at 36.50 mg/L (reference range, <5 mg/L). Computed tomography of the abdomen confirmed acute appendicitis (Fig. 1).

**Figure 2:**
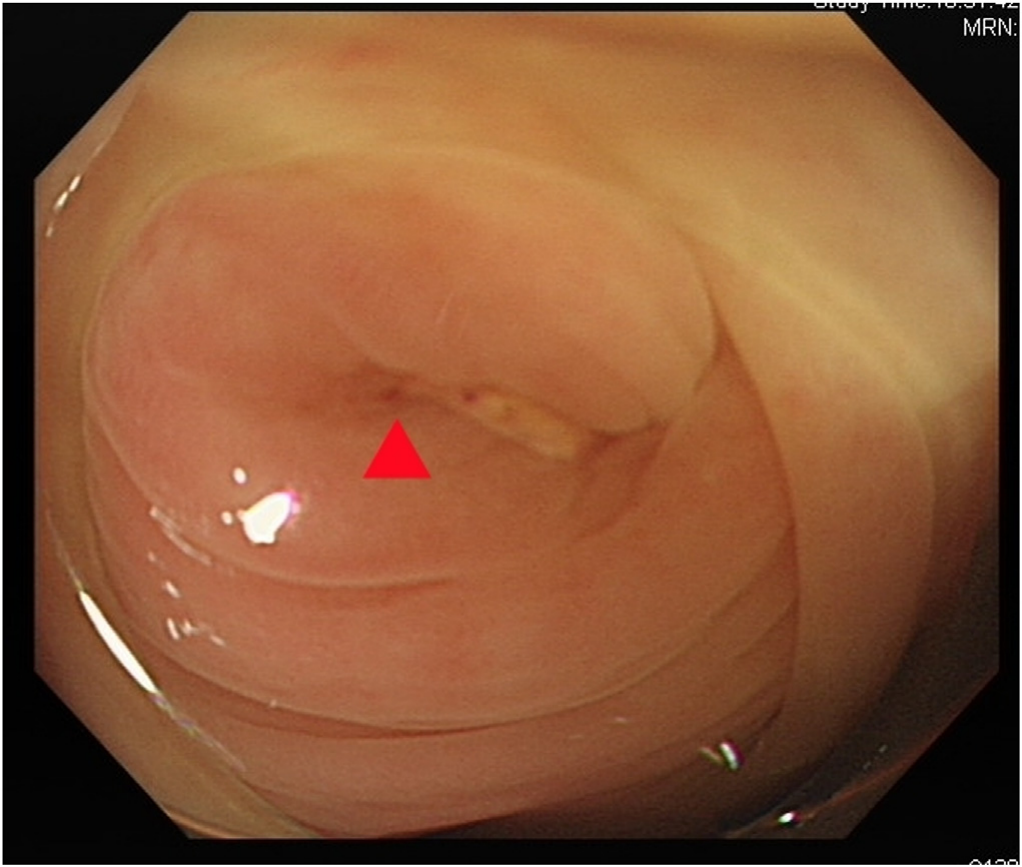
Colonoscopic image showing congested and edematous appendiceal orifice (*red arrowhead*) with purulent discharge to the right of the arrowhead.

**Figure 3:**
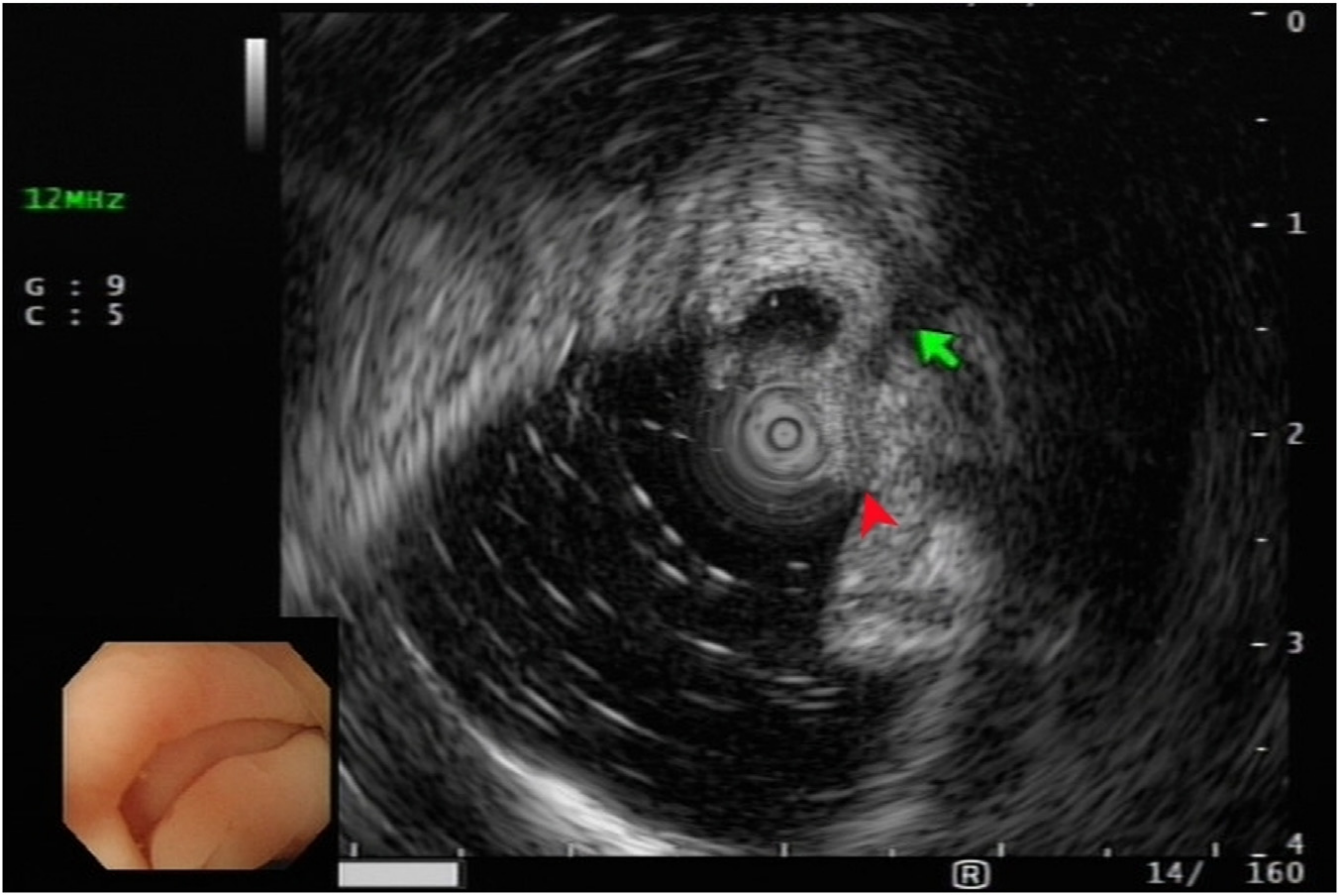
Endoscopic ultrasound image displaying appendiceal orifice (*red arrowhead*) and distended appendix (*green arrow*).

**Figure 4:**
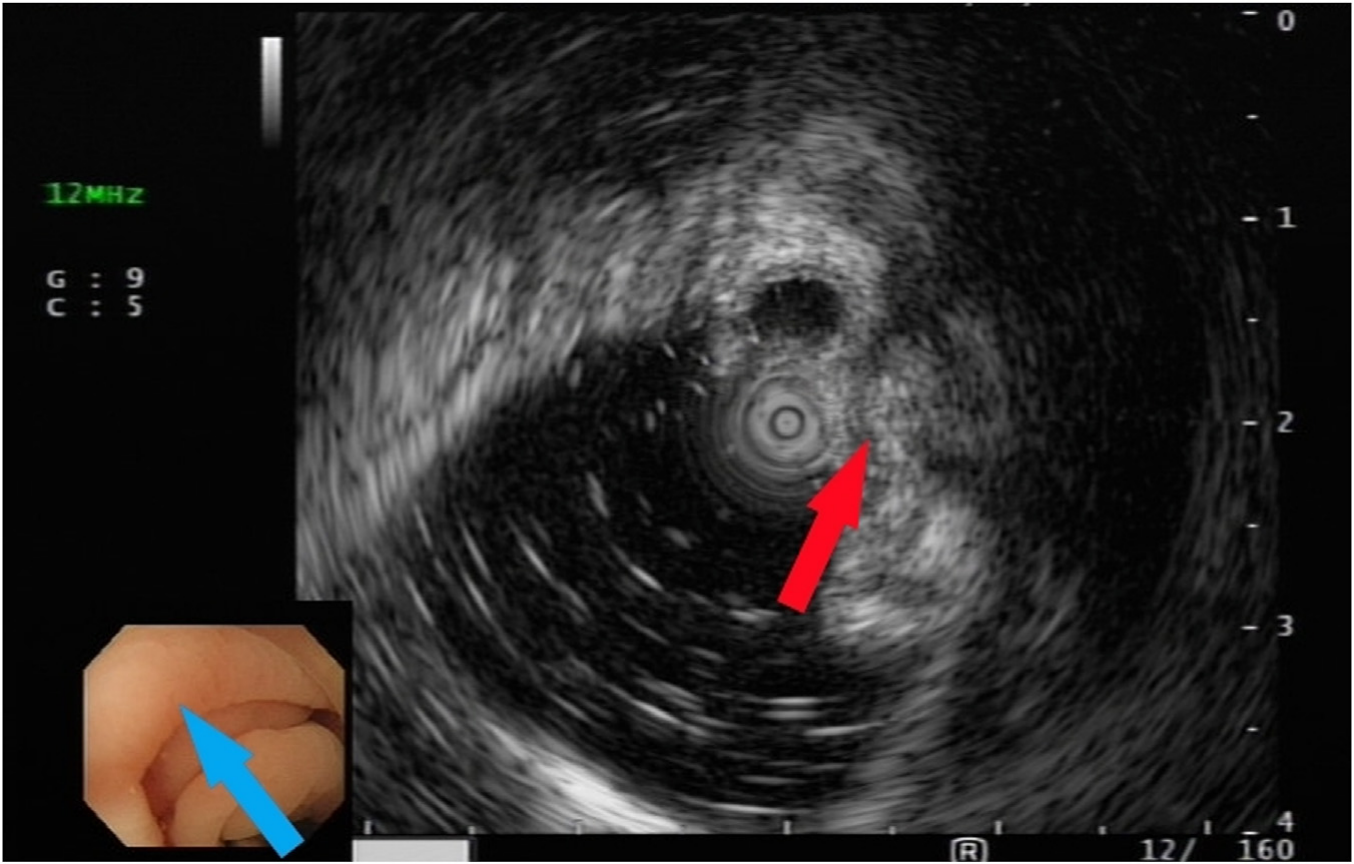
Endoscopic ultrasound image identifying the orientation of the appendiceal lumen relative to the appendiceal cavity (*red arrow*) and guiding the corresponding direction for appendicoscopic intubation (*blue arrow*).

**Figure 5:**
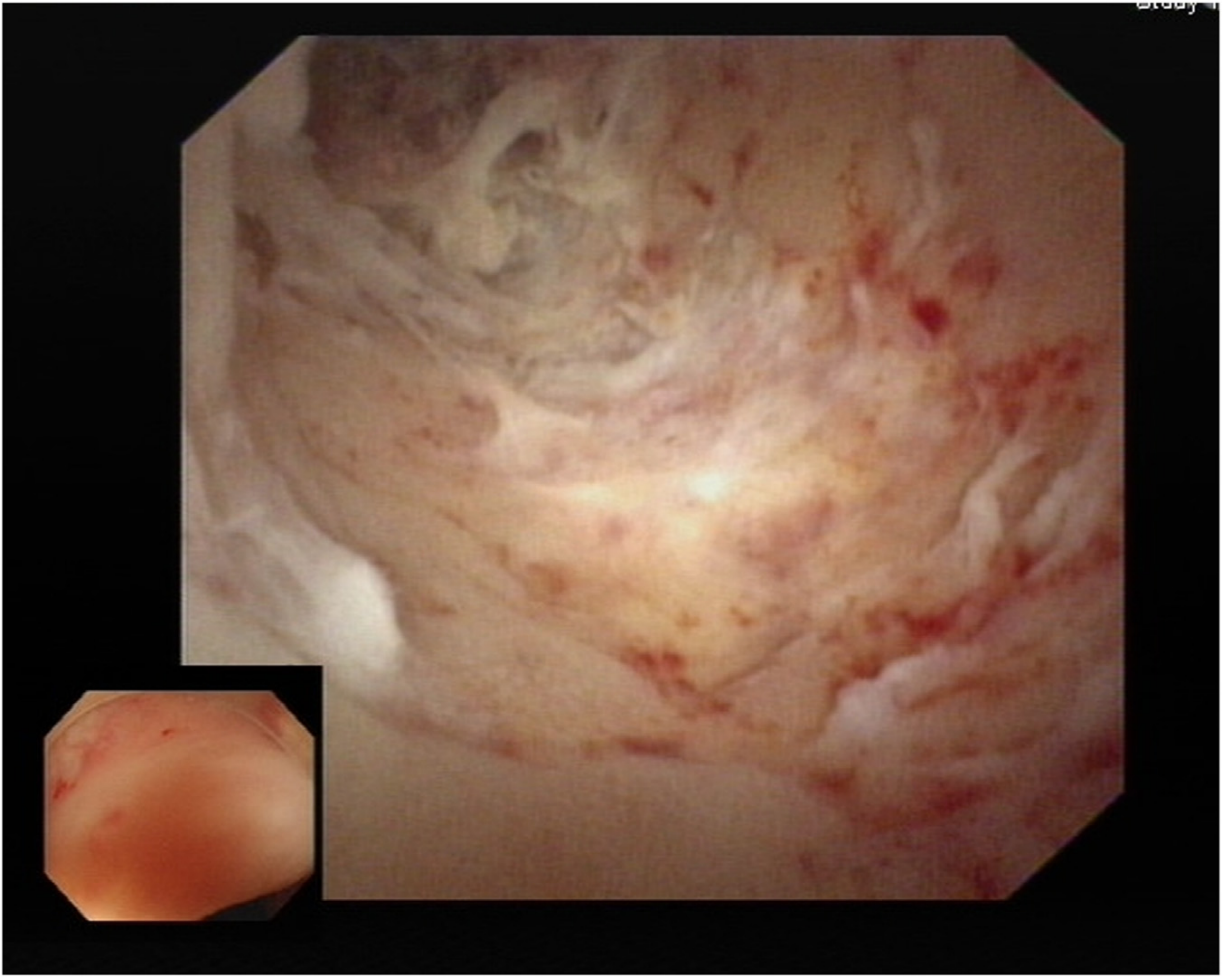
Appendicoscopy revealing inflamed mucosa and appendiceal cavity with purulent exudate.

**Figure 6:**
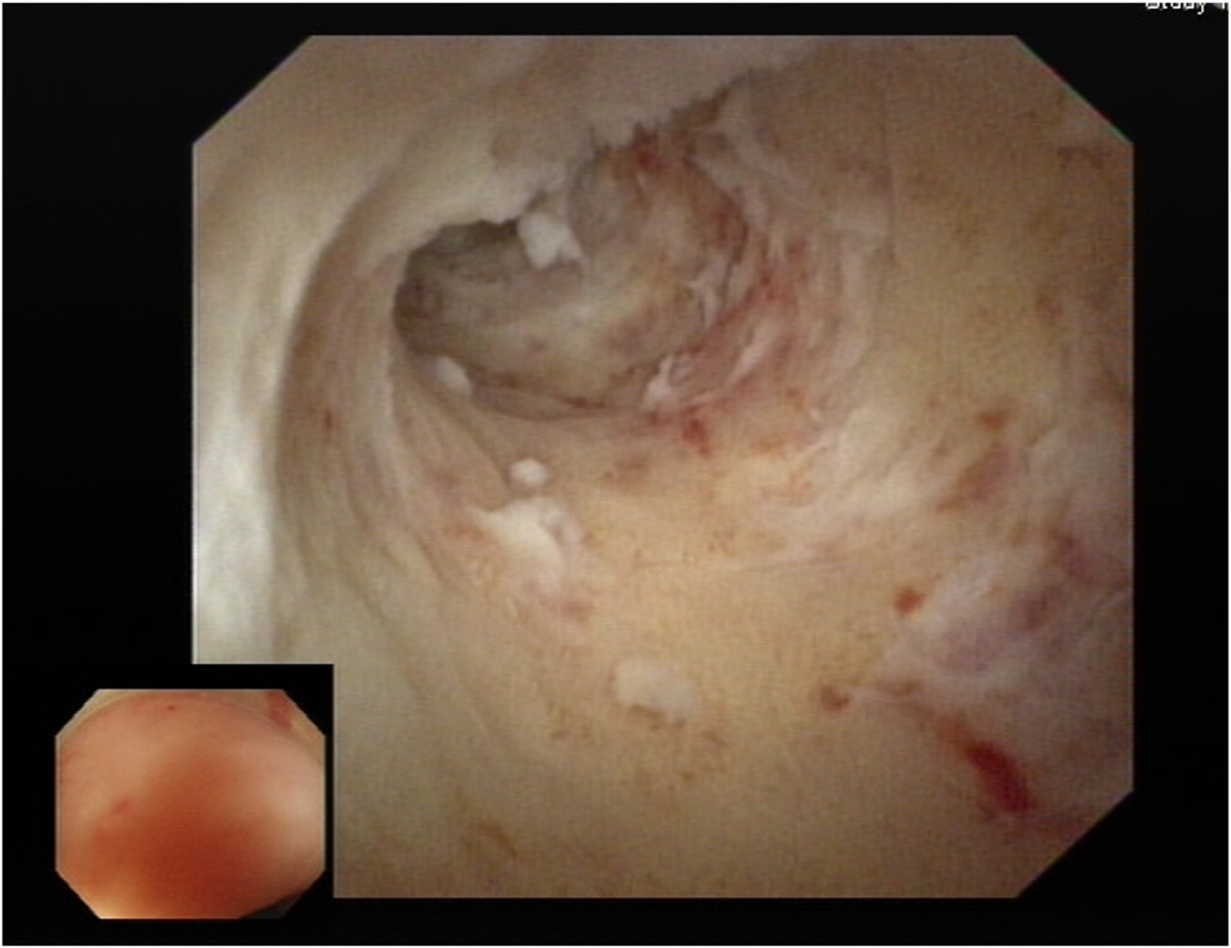
Appendicoscopy showing the tip of the appendiceal cavity with erythematous mucosa lining.

After we discussed the treatment options, including appendectomy, antibiotics alone, and endoscopic therapy, with the patient, she decided to undergo EAT. Colonoscopy revealed an inflamed appendiceal orifice with pus exudate (Fig. 2). Subsequent EUS with a 12-MHz frequency miniprobe (Olympus UM-2R, Tokyo, Japan) demonstrated an inflamed appendix with surrounding inflammation (Fig. 3). Using EUS, the endoscopist could identify the orientation of the appendiceal tip (Fig. 4), thereby facilitating smooth intubation. By the use of this information, a single-use cholangioscope was introduced through the therapeutic channel of the colonoscope. Assisted by EUS, the cholangioscope was skillfully and smoothly intubated into the appendiceal orifice and advanced into the lumen (Fig. 5). Appendicoscopy revealed erythematous mucosa with patchy hemorrhagic areas and fibrinous exudates, indicative of an inflammatory process consistent with appendicitis. The lumen appeared patent, with no evidence of obstruction or foreign material (Fig. 6). The inflammation was managed through repeated irrigation with 500 mL saline and 200 mL metronidazole solution (0.5 g/100 mL) until the pus was cleared. This approach improved visualization of the mucosa. After the procedure, the patient experienced significant pain relief and had an uneventful postprocedural recovery. Both her leukocyte count (6.1 × 10^9^/L) and neutrophil percentage (71.2%) normalized, and her high-sensitive C-reactive protein level improved to 8.97 mg/L 2 days after the procedure. She was subsequently discharged with a 5-day course of cefixime. At her 3-month follow-up visit, she remained free of adverse events or recurrence and expressed satisfaction with the procedure, particularly appreciating the avoidance of surgical intervention and the prompt resolution of her symptoms. The report was reviewed and approved by the Institutional Review Board of Xiamen Chang Gung Hospital.

## Discussion

Advancements in endoscopic technology have made nonsurgical management of acute appendicitis more feasible, as evidenced by the development of techniques such as endoscopic retrograde appendicitis therapy (ERAT).^4^ Whereas ERAT provides a minimally invasive alternative to surgery, it requires fluoroscopic imaging for retrograde appendicography, which offers only indirect visualization with limited guidance. By contrast, appendicoscopy with the use of a cholangioscope allows for direct visualization of the appendiceal lumen, facilitating both diagnostic and therapeutic interventions. However, successful EAT is highly dependent on the intubation of the appendiceal orifice, which can be challenging because of anatomic variations in appendiceal length, volume, and orientation.^5^ EUS assistance has served as a valuable tool in overcoming these challenges, enhancing the maneuverability and potentially shortening the procedural time of the appendicoscopy by eliminating the need for blind intubation. This approach is particularly advantageous for endoscopists who are in the early stages of mastering appendicoscopy, as it provides assistance that may improve the success of the technique and efficiency of the procedure. This case report is limited by its single-patient nature, requiring further studies to confirm the generalizability and long-term outcomes of EUS-assisted appendicoscopy.

## Disclosure

All authors disclosed no financial relationships.
